# BLINK enables ultrafast tandem mass spectrometry cosine similarity scoring

**DOI:** 10.1038/s41598-023-40496-9

**Published:** 2023-08-18

**Authors:** Thomas V. Harwood, Daniel G. C. Treen, Mingxun Wang, Wibe de Jong, Trent R. Northen, Benjamin P. Bowen

**Affiliations:** 1 Environmental Genomics and Systems Biology Division, The DOE Joint Genome Institute, Lawrence Berkeley National Laboratory, One Cyclotron Road, Berkeley, CA 94720 USA; 2 Department of Computer Science and Engineering, University of California Riverside, 900 University Avenue, Riverside, CA 92521 USA; 3 Computational Chemistry, Materials and Climate Group, Computational Research Division, Lawrence Berkeley National Laboratory, One Cyclotron Road, Berkeley, CA 94720 USA

**Keywords:** Mass spectrometry, Metabolomics

## Abstract

Metabolomics has a long history of using cosine similarity to match experimental tandem mass spectra to databases for compound identification. Here we introduce the Blur-and-Link (BLINK) approach for scoring cosine similarity. By bypassing fragment alignment and simultaneously scoring all pairs of spectra using sparse matrix operations, BLINK is over 3000 times faster than MatchMS, a widely used loop-based alignment and scoring implementation. Using a similarity cutoff of 0.7, BLINK and MatchMS had practically equivalent identification agreement, and greater than 99% of their scores and matching ion counts were identical. This performance improvement can enable calculations to be performed that would typically be limited by time and available computational resources.

## Introduction

Tandem mass spectrometry has become a critical component of both targeted and untargeted metabolomics experiments. Comparing fragmentation spectra between authentic standards and experimental data is central to making high confidence assignments in targeted metabolomics experiments. Untargeted experiments depend on matching experimental MS2 spectra against databases such as GNPS^1^, Metlin^2^, and MassBank^3^. Although different approaches have been developed^4,5^**,** this is still conventionally done by aligning fragment ions that share the same mass-to-charge ratio (*m/z*) and calculating the cosine similarity of their intensities. As a consequence of early mass spectrometers having low resolution, integer binning was a sufficient alignment step for comparing fragmentation spectra^6^. But as the resolution of mass spectrometers grew, so too did the sophistication of alignment algorithms necessary for high confidence identification and similarity scoring. Many of these approaches use iterative operations to compare individual fragment ions between reference and experimental spectra, which is inherently slower than vectorized operations.

To this end we introduce BLINK, an approach that enables scoring of fragmentation spectra using sparse matrix operations without the drawbacks of traditional *m/z* binning-based approaches. Rather than multiplying two matrices of binned fragmentation spectra directly, we use a uniform kernel to link together bins that are within machine noise tolerance, thereby vectorizing the previously costly alignment step. Matching ion counts can be approximated via the same methodology. Here we describe BLINK’s implementation and present a comparison between BLINK and other high performance similarity algorithms like GreedyCosine in MatchMS and Flash entropy^7,8^.

## Results and discussion

**Figure 1:**
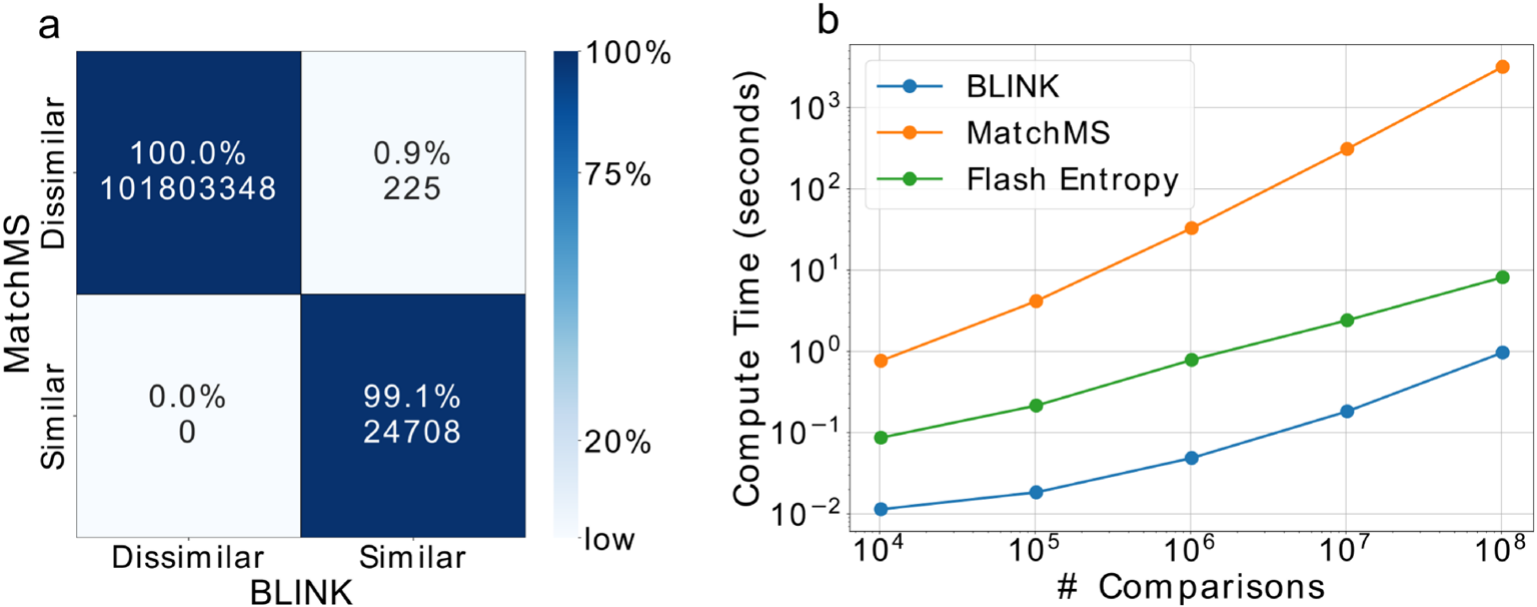
Evaluation of timing and agreement comparing BLINK cosine scoring to MatchMS and Flash entropy. (**a**) Modified confusion-matrix of spectral similarity using BLINK and MatchMS on over 100 million comparisons where “similar” results had high scores and high matching ions. In addition to raw counts, the percentages of counts normalized by MatchMS values are shown. (**b**) Median cosine scoring runtimes for BLINK (blue), MatchMS (orange) and Flash entropy (green) across 3 replicates. BLINK and Flash entropy scoring was performed without GPU acceleration.

For this study, we focused our score equivalence comparison on MatchMS because it is a high-performance, widely used, and well-supported Python package with overlapping use cases with BLINK. Flash entropy was only benchmarked against BLINK for speed, because the underlying similarity calculation used is mathematically distinct from cosine similarity^4^. To assess BLINK’s agreement with canonical cosine-based scoring approaches, we used tandem mass spectra sampled from an aggregate of all publicly available GNPS libraries. Using both MatchMS and BLINK, two randomly selected sets of MS/MS spectra were scored against each other. Spectra were classified as “similar” if their cosine score was ≥ 0.7 and matching ions were ≥ 6. All 24,708 spectra classified as similar by MatchMS were also similar using BLINK, however, 225 spectra were only classified as similar by BLINK (Fig. 1a). The spectra classified as similar by both BLINK and MatchMS had raw scores that varied by a mean value of 0.0004 and matching ion counts that varied by a mean value of 0.06. The deviation in scores and counts is due to BLINK’s alignment-free approach, which factors all ions within tolerance into the scoring and counting, rather than selecting only one.

While the results were similar, BLINK was much faster. Importantly, BLINK scales more favorably with high numbers of comparisons. BLINK can perform 1e8 comparisons 3,312 times faster than MatchMS (0.96 s for BLINK vs 53 min for MatchMS) (Fig. 1b). Additionally, BLINK was 8 times faster than Flash entropy. This difference is because standard tools such as MatchMS rely on loop-based, pairwise alignment procedures for matching ions within machine noise tolerance during scoring. BLINK circumvents this computationally expensive fragment alignment step. In addition, rather than iteratively scoring pairs of spectra, BLINK simultaneously scores all pairs of spectra via sparse matrix operations. Searches using tandem mass spectra for chemical analogs typically combine cosine scoring with shifting the spectra by their difference in precursor *m/z*. BLINK also has the capability of performing analog searches by precursor *m/z* difference or any user defined mass difference.

Furthermore, depending on the number of comparisons and hardware specifics, the scoring speed can be increased by an additional 5–10 times if using GPU acceleration. This speedup can enable investigators to perform high-throughput calculations that would otherwise be prohibitively time intensive. For example, NIST20^9^ currently has over 1.3 million spectra and a typical metabolomics data set can have 10,000 features. Extrapolating from the speed tests in Fig. 1b, performing a complete database search would take 4.5 days with MatchMS, and this is reduced to 3.3 min using BLINK. Our benchmarking was performed without database indexing to more directly compare algorithm performance, however, pre-indexing of library spectra prior to scoring could also be utilized to further decrease compute time.

**Figure 2:**
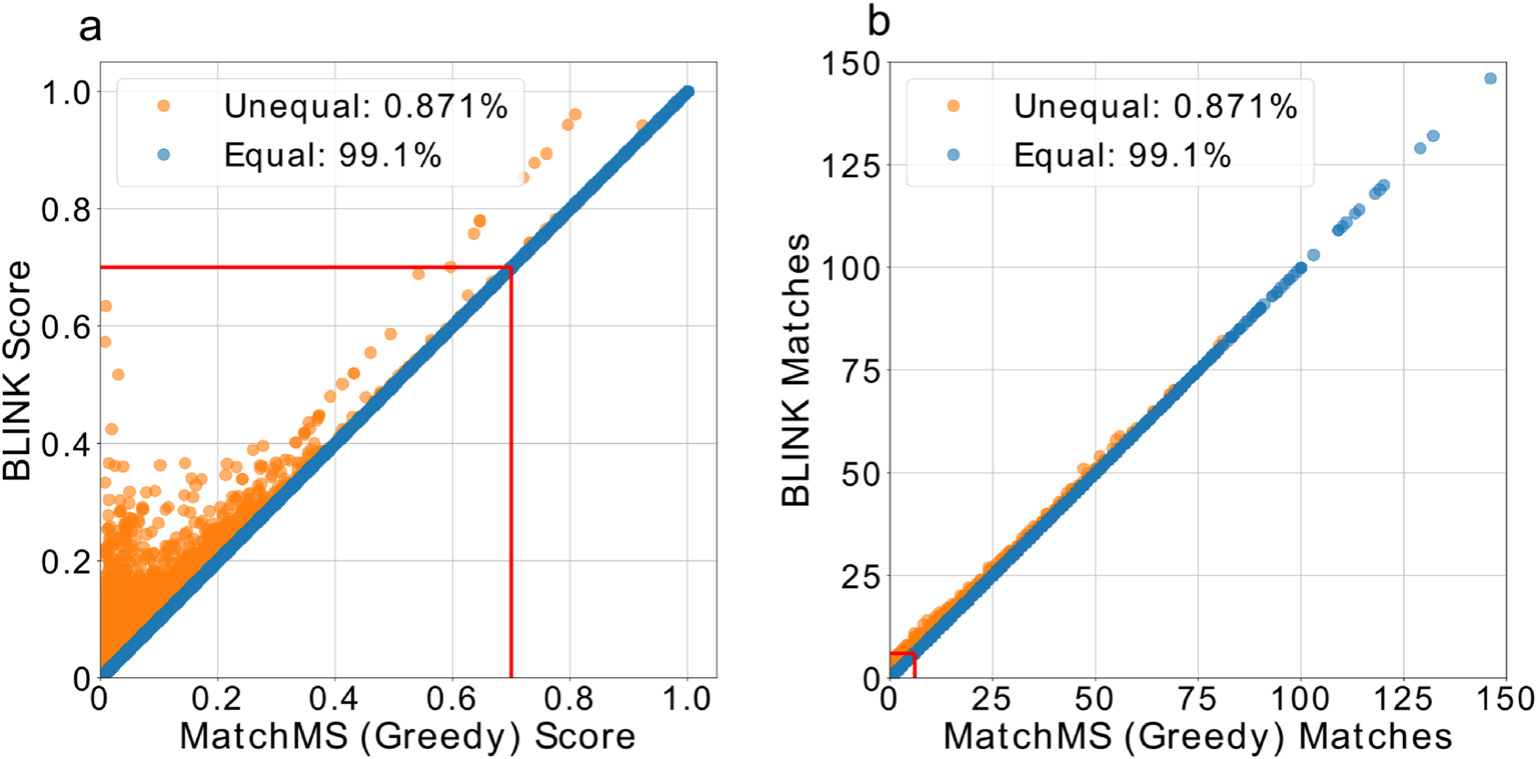
Direct comparison of scores (**a**) and matching ions counts (**b**) between BLINK and MatchMS. Score and match thresholds of 0.7 and 6 respectively are represented by red lines. Comparisons with exact matching ion counts and scores within 0.001 are shown in blue. The remaining comparisons are shown in orange.

There are some limitations to BLINK’s alignment-free approach. Because all ions within tolerance are factored into the cosine score, scores calculated using BLINK diverge from precise, loop-based implementations as tolerance increases, resulting in higher scores and matches (Fig. 2). Therefore, traditional tools may be a more appropriate choice for data generated by low resolution mass spectrometers that require a wide tolerance window. However, given trends in mass spectrometry hardware development, this limitation will likely become less relevant over time as higher resolution machines are continually developed^10^. One viable strategy for use cases that require 100% agreement with conventional approaches is to recalculate the top BLINK hits using precise methods, maintaining much of the performance advantages of BLINK’s alignment-free approach while producing identical scores. For instance, to recalculate the 24,933 BLINK hits from Fig. 1a would take less than 10 s with MatchMS. We envision that the speed-up using BLINK will enable scientists to efficiently make chemical assignments as the size of MS2 databases continues to grow, particularly in-silico databases uncoupled from the availability of new authentic standards and instrument throughput ^11,12^.

**Figure 3:**
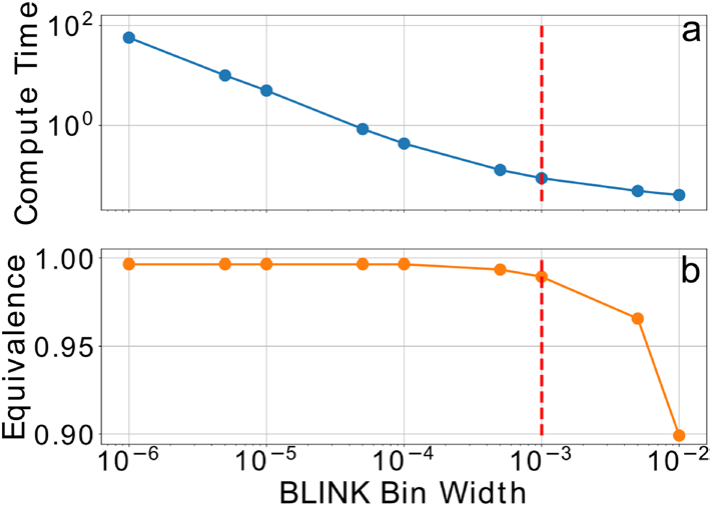
BLINK compute time in seconds per ten million comparisons (**a**) and equivalence of scores to MatchMS results (**b**). Recommended bin width of 0.001 is represented by a dashed red line.

Depending on the BLINK bin width used, compute time and agreement with MatchMS vary (Fig. 3.). While scoring speed increases as bin width increases, using a bin width larger than 0.001 results in diminishing gains in performance. Additionally, while equivalence to MatchMS remains constant when bin width is smaller than 0.0001, there is a significant increase in compute time. The divergence in scores between MatchMS and BLINK as bin width increases can be explained by the binned fragment *m/z* values of two spectra being compared being within tolerance to each other while the original values were not, or vice versa. For use cases that require high agreement to conventional approaches, the bin width should be informed by the reliable accuracy of the mass spectrometer used to collect the experimental tandem mass spectra.

## Methods

### Implementation

**Figure 4:**
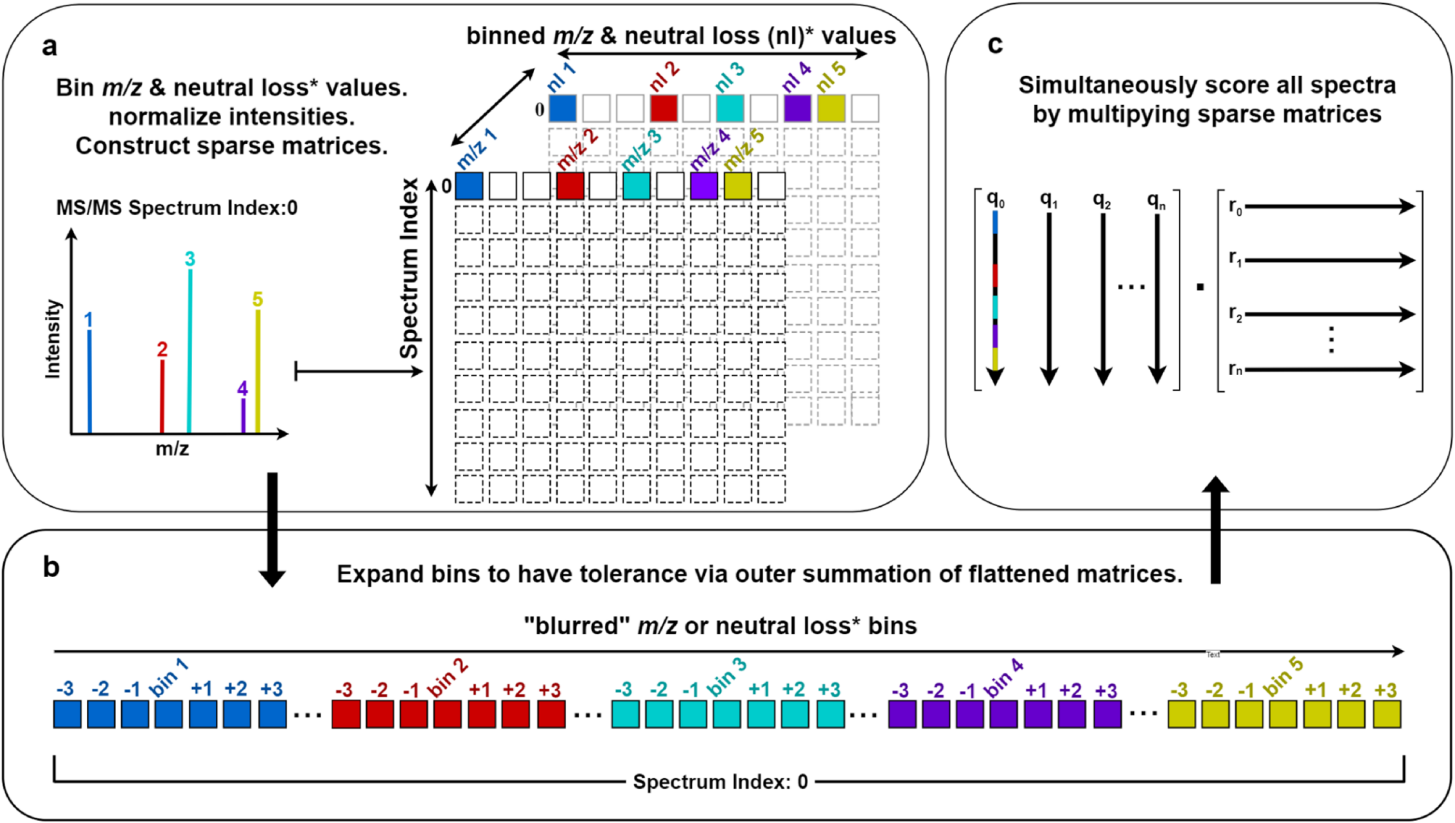
Workflow model illustrating the general methodology used to calculate cosine similarity scores and matching ion counts using BLINK.

Typically, two fragmentation data files, a query and a reference, are used as input. The files are parsed and the fragmentation spectra are represented as lists of *m/z* and intensity arrays with associated metadata. To discretize the spectra, *m/z* values are first converted to integer-bins based on the user defined bin width (default is 0.001 Da). These values can optionally be shifted by the precursor *m/z* or other user defined mass for analog searches. Intensity arrays in each spectrum are then unit-vector normalized. Each set of processed spectra is converted into two sparse matrices. The first contains fragment intensities, and the other fragment counts. Each sparse matrix is constructed such that rows are *m/z* bins and columns are the spectrum index (Fig. 4a).

Prior to scoring, the *m/z* bins are expanded (i.e. blurred) by distributing intensities and summing fragment *m/z* bins with a tolerance-window wide array, referred to here as BLINK’s kernel. This allows for *m/z* bins to be linked between spectra within a tolerance parameter (default is 0.01 Da). It is only necessary to perform this computation on one set of spectra, so the kernel is used to transform the smaller of the two (typically the query) (Fig. 4b).

Each pair of sparse matrices are resized such that they contain an equal number of rows, and one of the matrices is transposed. The two matrices are multiplied, generating the score matrix (Fig. 4c). When performed on the matrices containing intensity data, this step simultaneously computes the cosine scores for each combination of spectra. Fragment counting is based on the same algorithm, but uses the fragment count matrices as input. Scoring operations can optionally be performed with GPU acceleration.

### Speed benchmarking and similarity agreement

Calculations were done using an exclusive CPU node on the Perlmutter supercomputer at NERSC. Each CPU node is equipped with 512 Gb of DDR4 RAM and dual AMD EPYC 7763 64 × core processors. Two sets of spectra were randomly sampled from an aggregate of all GNPS library spectra (All-GNPS) as of January 17th 2022 ranging in size from 1e^2^ to 1e^4^. Each subset of spectra was sampled independently without replacement. Scoring was performed using tolerance values of 0.01 Da and 0.009 Da for BLINK and MatchMS respectively. Tolerance for Flash entropy was similarity set to 0.009 Da. The BLINK bin width parameter was set to 0.001 Da. BLINK’s true tolerance is equivalent to the tolerance parameter subtracted by the bin width, hence the difference in tolerance values used for the three algorithms. To improve scoring behavior of all algorithms, the spectra were filtered to remove noise ions and the intensity values were scaled by their square-root. Fragment ion noise filtering was accomplished by removing ions that were < 1% of base peak intensity and ions with *m/z* values greater than the precursor *m/z*. Additionally, all ions with intensity values of 0 were removed from the spectra.

Progressively larger sets of spectra were sampled and scored with 3 replicates and their median calculation timings with MatchMS, BLINK, and Flash entropy were reported in Fig. 1b. The modified confusion-matrix reported in Fig. 1a was generated using 1e^8^ scores from the first replicate of the speed benchmark. Spectra were classified as similar if their score was ≥ 0.7 and matching ions were ≥ 6. These values were chosen because they are commonly used in the field.

### Mean score and matching ion count differences

The same set of scores used to construct the confusion-matrix were used to calculate the mean score and matching ion count differences. Differences of the true positive scores (classified as similar by both MatchMS and BLINK) were calculated by subtracting the BLINK scores and counts by their MatchMS counterpart.

### Direct score and matching ion count comparison

Two sets of spectra were sampled from the publicly available Berkeley Lab spectral library in GNPS to make a total of 10 million comparisons. Pre-processing of spectra was performed as described above for speed benchmarking prior to scoring. Scores were considered equivalent if the difference was less than 0.001, and matching ion counts had to be identical. For this comparison, a bin width of 0.001, a BLINK tolerance of 0.01, and a MatchMS tolerance of 0.009 was used.

### Speed benchmarking and similarity agreement across bin widths

The same 10 million comparisons used to generate the direct score and matching ion count comparison reported in Fig. 3 were recalculated across nine bin widths, from 10e^−6^ to 0.01, maintaining a constant BLINK tolerance value of 0.01. The largest bin width of 0.01 was chosen because BLINK bin width cannot exceed tolerance.

## Conclusion

Comparison of fragmentation spectra has become a primary step in both targeted and untargeted metabolomics workflows. Using an alignment-free and vectorized approach, BLINK is able to compute cosine similarity scores of tandem mass spectrometry data faster than previously possible. Currently, our implementation is limited to calculating cosine similarity, however, adapting this framework to other similarity measures like spectral entropy^4^ or SIMILE^13^ to enable performance gains is a promising area of future work.

## Data Availability

BLINK is implemented in Python3 and is published under a modified open-source license. Code and license are available on Github: https://github.com/biorack/blink.
